# Parenting practices in occupational justice lens in the post-genocide context: more than 31 years after a genocide against the Tutsi in Rwanda

**DOI:** 10.3389/fresc.2025.1498419

**Published:** 2025-06-30

**Authors:** Emmanuel Biracyaza, Samuel Habimana

**Affiliations:** ^1^ School of Rehabilitation, Faculty of Medicine, Université de Montréal, Montreal, QC, Canada; ^2^ Centre for Interdisciplinary Research in Rehabilitation of Greater Montreal (CRIR), Montreal, QC, Canada; ^3^ Department of Administration and Research, Rwanda Resilience and Grounding Organization (RRGO), Kigali, Rwanda; ^4^ Department of Social Work and Social Ecology, School of Behavioral Health, Social Welfare, and Social Research Program, Loma Linda University (LLU), Loma Linda, CA, United States

**Keywords:** parenting practices, occupation, post-conflict, genocide, occupational justice

## Purpose of the study

This paper explores parenting as a central yet often overlooked social occupation in post-genocide Rwanda. Using the lens of occupational justice, it examines how long-term trauma has impacted parenting practices and proposes collective, community-based strategies to support and empower families. It seeks to address a gap in occupational science by advocating for the integration of culturally grounded, community-driven, and participatory approaches to support Rwandan families in reclaiming their parenting roles. By centering the voices and experiences of Rwandan scholars, parents, and communities, the article underscores the transformative potential of parenting in healing, reconciliation, and sustainable community rebuilding in post-conflict societies.

## Context

From the occupational science (OS) perspective, parenting is conceptualized as a meaningful daily and essential occupation that involves caregiving, protecting, nurturing, teaching, and guiding children (1). It is not only a personal or familial responsibility but also a fundamental right, one that must be upheld by ensuring parents have fair access to emotional support, community engagement, and social resources especially for those impacted by trauma such as genocide (2). Research shows that parenting as an occupation plays a crucial role in promoting psychosocial wellbeing of parents, children, and communities (1, 3). In post-conflict environments, such as Rwanda following the 1994 genocide against the Tutsis, this role becomes even more critical. Although a study among victims related to genocide stated that mothering became a source of resilience (4), this holocaust inflicted profound and lasting wounds that continue to affect parenting practices, family dynamics, and the overall well-being of the current generations and upcoming generations (5). Given this context, our paper seeks to advance the discourse on how occupational science can address the injustices that trauma imposes on parenting—such as social exclusion, stigma, and limited access to support systems. We argue that OS offers valuable insights and frameworks to support affected families and restore their parenting capacities. More specifically, this article explores how engaging in the occupation of parenting can become a vehicle for social healing, community resilience, and long-term recovery in the aftermath of mass violence.

Genuinely, we are grateful for the opportunity to write for several reasons. Firstly, our up-to-date research projects focus on strengthening parenting practices in the context of post-genocide Rwanda, aligning with the critical themes situated in occupation angles. Second, through more than a decade of fieldwork with affected populations, we have developed insights that underscore the enduring effects of trauma on parenting roles. Now, over three decades since the genocide, it is more urgent than ever to explore how trauma continues to shape parenting occupations and the upbringing of subsequent generations (6, 7). Hence, our research and field experiences on parenting and psychosocial health in post-genocide advocate strongly for adopting an occupational justice framework to tackle these challenges, offering a promising path toward fostering resilient and healthy family systems (6, 7).

OS recognizes parenting as an essential occupation—a daily, meaningful activity that is crucial for individual and community well-being (1, 8–11). Yet in the aftermath of the genocide in Rwanda, many parents many parents including survivors, former perpetrators, and their descendants face deep challenges that hinder their ability to fully engage in this role. The long-term impacts of trauma can disrupt emotional regulation, weaken bonds between parents and children, and impair caregiving abilities, often resulting in cycles of parenting struggles passed from one generation to the next (12, 13). In this context, the occupational justice framework provides an important lens through which to understand and address these disruptions and deprivation affecting parenting role, as it highlights how contextual and structural factors such as poverty, access to health services, trauma, and social exclusion can limit access to meaningful occupations, including parenting (14, 15).

## Occupational justice and parenting in post-conflict settings

Many studies in Occupational Science (OS) and Occupational Therapy (OT) tend to prioritize clinical interventions within medicalized environments, which has contributed to the limited attention given to parenting as an occupation especially in post-conflict settings (16, 17). This trend reflects a historical emphasis on individual-level rehabilitation focused on restoring physical or cognitive function within healthcare institutions. As a result, broader social roles such as parenting, which are essential for community and societal well-being, have been overlooked (18). Moreover, OS and OT have often neglected everyday occupations like parenting, particularly in under-researched and structurally complex contexts such as post-conflict communities, where environmental and social conditions heavily influence parenting roles. Although parenting is increasingly recognized as a significant occupation, it remains underrepresented in occupational literature compared to more conventional rehabilitative topics (3, 19). This gap is especially pronounced in sub-Saharan Africa (SSA), where OS and OT are still emerging disciplines despite the region facing numerous occupational challenges (20). Within these contexts, parenting, particularly as a socially embedded and meaningful occupation has received minimal scholarly attention, especially beyond early childhood care. The majority of studies emphasize early childhood development and parental training in caregiving, frequently marginalizing fathers and neglecting the framing of parenting as an occupational process (21–23).

In Rwanda, where families have been disrupted and social structures weakened, rehabilitating parenting practices is essential but remains underexplored in mainstream clinical fields rather than as a social occupation (24). Additionally, the dominance of the medical model: the medical model of rehabilitation prioritizes diagnosing and treating physical or mental impairments. Consequently, this model often overlooks the social, cultural, and occupational dimensions of recovery, such as parenting, caregiving, and community involvement (14, 25). Parenting, especially in post-conflict contexts, involves rebuilding trust, creating safe environments for children, and addressing trauma—tasks that go beyond clinical rehabilitation and fall into social and community development areas. Thirdly, challenges in post-conflict contexts such as Rwanda following the genocide, parenting is shaped by profound trauma, loss, and disrupted family systems. These contexts necessitate the application of a distinct and innovative participatory occupational justice framework (POJF) (26) to promote occupations through occupational science lens for assisting parents and empowering their communities (25, 27, 28). However, research often prioritises immediate needs including physical rehabilitation, rather than focusing on the long-term occupation of parenting and its role in rebuilding healthy, resilient families. Fourthly, the lack of frameworks for occupational justice: parenting as an occupation, especially in post-conflict scenarios, is rarely framed as an important daily activity. It is often treated as a social or developmental issue rather than an occupation (29). To our knowledge, this gap means that the intersection of parenting, trauma recovery, and community empowerment in occupational perspectives is often neglected.

## Culturally sensitive approaches to parenting interventions

Parenting interventions in post-genocide Rwanda should be both culturally sensitive and grounded in the lived experiences of local contexts for promoting sustainable outcomes. While international researchers have contributed significantly to the field, it is critical to center the voices of Rwandan scholars and community members in developing strategies for strengthening parenting practices (30–32). Too often, external solutions have failed to address the specific needs of post-genocide communities due to not fully prioritising the voices of parents. These challenges lead to a mismatch between interventions and local realities. Further, it is important to adopt bottom-up approaches that emphasizes community-driven, participatory processes rooted in local knowledge and lived experiences. Unlike top-down models that impose externally designed interventions, bottom-up approaches in occupational science prioritize the empowerment of individuals and communities to identify their own needs, define meaningful occupations, and co-create solutions that are contextually and culturally relevant (33). This orientation aligns with human rights-based and occupational justice paradigms, which emphasize self-determination, inclusion, and agency (34, 35). It is important to clarify that while the term “bottom-up” can have different meanings across disciplines (e.g., in systems theory or policy studies), within occupational science it specifically refers to approaches that emerge from the ground up—valuing the priorities and strengths of the people involved over imposed service delivery models. This distinction is especially critical in post-conflict settings like Rwanda, where externally designed programs may risk overlooking the nuanced realities of trauma, culture, and social reconstruction. These approaches involve local stakeholders in the design and implementation of parenting interventions, ensuring that solutions are rooted in the unique social and cultural context of Rwanda (7, 30). Therefore, using participatory research approaches that prioritise collaboration with the communities and their members, parenting as an occupation can be supported and empowered (36).

## The role of parenting in healing and reconciliation

The dominance of the medical model, historical emphasis on individual-level reintegration, and limited funding for social interventions contribute to this gap (16). In post-genocide contexts like Rwanda, where OS is relatively new (established around 2015), this issue is particularly pronounced. The aftermath of the genocide has left profound trauma and disrupted family structures, making the role of parenting in community rebuilding even more critical. However, the nascent state of OS in Rwanda means that frameworks and research addressing the intersection of parenting, trauma recovery, and community rehabilitation are still developing. The lack of focus on these areas further exacerbates the challenge of addressing long-term recovery needs, highlighting the urgent need for integrating social occupations into rehabilitation research and practice.

In light of these considerations, and to concur with previous studies and recommendations to elevate the voices of Rwandan scholars and their communities (5), we would also like to underline the importance of supporting future research led by local academics and integration of innovative approaches that encourages collective efforts and community empowerment for sustainable social transformation. As noted, much of the research on Rwanda has historically been conducted by international scholars, often overlooking the perspectives of those most impacted. This practice sometimes referred to as “helicopter research,” involves researchers collecting data without contributing lasting benefits to the community (37). In Rwanda, families are at the heart of community rebuilding, and by strengthening the capacity of parents to provide emotional support, guidance, and stability, we can foster greater social cohesion. This aligns with the principles of occupational justice, which advocate for providing individuals with the resources and support they need to participate wholly in their communities.

For parents in Rwanda, the ability to engage in meaningful occupations, such as raising their children, is an essential part of rebuilding their lives after trauma. By addressing the occupational injustices that limit their capacity to parent effectively, we can empower families and communities to overcome the long-term effects of the genocide. Besides, supporting parents to re-engage in their parenting role can help rebuild identity, strengthen families, and foster community resilience. This empowerment is critical not only for individual families and communities but also for the broader goal of national reconciliation, family promotion and child development. At the regional level, particularly in areas like SSA (16), advancing research in occupational sciences to include social occupations like parenting role will illuminate local needs and support more effective, community-driven development strategies. By focusing on region-specific needs and contexts, we can develop tailored strategies that address the unique aspects of parenting and community rebuilding in post-conflict settings. Such studies will not only contribute to the regional body of knowledge but also inform global practices and policies, ensuring that they are impactful.

## Conclusions

Parenting is not merely a personal responsibility; it is a critical social occupation that has far-reaching implications for community rebuilding and resilience. Despite its centrality, parenting has often been marginalized within rehabilitation science, which has traditionally prioritized clinical and individual-focused interventions. To achieve holistic and sustainable recovery, it is imperative that rehabilitation frameworks evolve to incorporate social occupations like parenting. This expansion allows for interventions that not only address individual trauma but also strengthen the foundational social structures necessary for cohesive communities. Drawing on the POJF, we advocate for inclusive, collaborative, and contextually grounded strategies that place communities, especially parents, at the center of designing and implementing parenting support. As the African proverb aptly states, “If you want to go fast, go alone; if you want to go far, go together.” This ethos reinforces the necessity of collective action in exploring and addressing occupational injustices stemming from historical and structural trauma. By giving priority, the voices of Rwandan scholars, caregivers, and communities, and grounding interventions in their lived experiences, we can foster culturally attuned and sustainable models of support. In doing so, parenting can be reclaimed as a transformative force for resilience, reconciliation, and generational healing in post-genocide Rwanda and beyond.

As Rwanda continues to navigate a path of growth and healing, we must reflect critically on whose voices are amplified in mental health research and what forms of parenting practices are respected or spurned. In a society striving for rapid progress, how can we ensure that parenting remains a central, supported occupation rather than an invisible burden in some families and communities?

We, therefore, leave readers and policymakers with these crucial questions:

• How can we create spaces where parenting is recognized and supported as a fundamental right and occupation essential to national healing?
• What systems must be in place to ensure parents especially those historically marginalized have sufficient resources, time, and community support to nurture their children meaningfully?
• How might we dismantle contextual and structural barriers that prevent parents from fully engaging in their caregiving roles?
